# Imaging fungal infections in children

**DOI:** 10.1007/s40336-015-0159-2

**Published:** 2016-01-25

**Authors:** Alfred O. Ankrah, Mike M. Sathekge, Rudi A. J. O. Dierckx, Andor W. J. M. Glaudemans

**Affiliations:** Department of Nuclear Medicine and Molecular Imaging, University Medical Center Groningen, University of Groningen, Hanzeplein 1, PO 9700 RB Groningen, The Netherlands; Department of Nuclear Medicine, University of Pretoria and Steve Biko Academic Hospital, Pretoria, South Africa

**Keywords:** Fungal infections, Children, FDG-PET, Hybrid imaging, Aspergillus, Candida

## Abstract

Fungal infections in children rarely occur, but continue to have a high morbidity and mortality despite the development of newer antifungal agents. It is essential for these infections to be diagnosed at the earliest possible stage so appropriate treatment can be initiated promptly. The addition of high-resolution computer tomography (HR CT) has helped in early diagnosis making; however, it lacks both sensitivity and specificity. Metabolic changes precede anatomical changes and hybrid imaging with positron emission tomography (PET) integrated with imaging modalities with high anatomical resolution such as CT or magnetic resonance imaging (MRI) is likely to detect these infections at an earlier stage with higher diagnostic accuracy rates. Several authors presented papers highlighting the advantages of PET/CT in imaging fungal infections. These papers, however, usually involve a limited number of patients and mostly adults. Fungal infections behave different in children than in adults, since there are differences in epidemiology, imaging findings, and response to treatment with antifungal drugs. This paper reviews the literature and explores the use of hybrid imaging for diagnosis and therapy decision making in children with fungal infections.

## Introduction

Fungal infections may be superficial, mucous, or invasive. Most superficial and some mucous fungal infections are easily accessible and can be diagnosed by clinical findings and microscopy. No diagnostic imaging modalities are necessary in those cases. On the other hand, in a few mucous and in most invasive fungal infections (IFIs), which may not be easily accessible, a proper diagnosis is essential and existing imaging techniques are of invaluable importance. In the past few decades, there has been a considerable increase in both the frequency and importance of IFIs [1]. This increase is directly related to the growing population of immunocompromised individuals, resulting from changes and advances in medical practice such as the use of intensive chemotherapy, immunosuppressive drugs, and growing stem cell transplantation possibilities. HIV and other diseases which cause immunosuppression have also contributed to this problem.

Children at risk of acquiring IFIs are children who undergo chemotherapy for malignancy, are treated with immunosuppressive drugs, have congenital or acquired immune deficiencies, and undergo hematopoietic stem cell transplantation (HSCT) or solid organ transplantation (SOT) [1, 2]. These diseases and/or treatment regimens represent “typical” risk factors for acquiring an IFI. Furthermore, neonates or children admitted to the intensive care unit (ICU) may also be at risk of developing IFIs. The children admitted to the ICU may or may not be neutropenic [3].The epidemiology and risk factors for IFIs are different for previously healthy children who have been admitted to the ICU compared to children with malignant or hematologic disorders [4]. The risk factors of acquiring an IFI for children admitted to the ICU include critical illness with long stay in ICU, prolonged use of antibiotics, abdominal surgery particularly with bowel anastomosis, insertion of central venous catheters or other foreign bodies into the blood vessels and low birth weight or prematurity in neonates [4]. A child on admission at the ICU may also have any of the typical risk factors for IFIs.

Candida and Aspergillus are the most common fungal agents responsible for IFIs. In children, invasive candidiasis occurs five times more frequent than invasive aspergillosis. On the contrary however, the mortality rate is 2.5–3.5 times higher for invasive aspergillosis compared to invasive candidiasis [4, 5]. The overall mortality rate attributable to patients with IFIs is 32 % [6]. The mortality rate for invasive aspergillosis is 70 % despite appropriate treatment, whereas it is between 20 and 30 % for invasive candidiasis [5, 7]. It is important to prevent these infections, and when they occur, IFIs must be diagnosed as early as possible and appropriate treatment should be initiated immediately.

## Pathogens

In general, fungi are classified into yeasts and molds with *Candida species* (*Candida sp.*) and *Aspergillus species* (*Aspergillus sp.*) being the most common type of each.

## Candida

Invasive Candida infection is the fourth most occurring blood stream infection in ICUs [8]. *Candida albicans (C. albicans)* is the most common cause of invasive candidiasis; however, in recent years, with the introduction of antifungal prophylaxis, there has been a reduction in the proportion of invasive candidiasis due to *C. albicans*, but an increase in cases of IFIs caused by less common *Candida sp.* such as *C. krusei*, *C. parapsilosis*, and *C. glabrata* amidst others which may vary in virulence and susceptibility to the antifungal drugs commonly used [8].

*C. glabratra* has emerged as an important pathogen in Northern Europe, the USA, and Canada, whereas *C. parapsilosis* is more prominent in Southern Europe, Asia, and South America. *C. parapsilosis* is less virulent than *C. albicans* and *C. glabrata*, and hence, it has lower mortality rates. Invasive candidiasis usually presents as candidemia with fever and sepsis. It may also occur as a blood culture negative syndrome such as disseminated (hepatosplenic) candidiasis with deep-seated infections in other organs such as bones, muscles, joints, and eyes, usually occurring in patients with hematologic cancer or disorders. These infections arise from an earlier or previously undiagnosed blood stream infection [8, 9].

## Aspergillus

Invasive aspergillosis is still a major cause of morbidity in severely immunocompromised patients. There are many species, and *Aspergillus fumigates* is the most common. Invasive aspergillosis presents with cough, dyspnea, pleuritic chest pain, and sometimes hemoptysis. It frequently occurs among patients with the typical risk factors and it is increasingly diagnosed in patients without the typical risk factors for IFIs in patients who are treated on the ICU with burns, trauma, or liver cirrhosis [4, 7, 10].

## Other fungal pathogens

While Candida and Aspergillus remain the two main fungi encountered in IFIs, less common organisms such as *Cryptococcus sp*., Histoplasmosis sp., Coccidiomycosis sp., Cryptococcus sp., *Murcomycosis sp*., and *Blastomycosis sp.* may also be etiological agents. Each of these has its specific characteristics. For example, *Histoplasmosis sp.* usually involves the reticuloendothelial system and frequently affects the adrenal glands, while *Cryptococcus sp*. may occur more commonly in HIV patients. These rare fungi have all been diagnosed in children [11, 12].

## Differences in IFIs between children and adults

Although children and adults are similarly vulnerable to IFIs, important differences exist in host responses, the capacity of immune reconstitution after chemotherapy, and comorbidity. These differences all influence the risk and outcome of IFIs [13]. In the neonatal period, neutrophils have impaired chemotaxis and bactericidal activity compared with older children and adults [14, 15]. Furthermore, T cell regeneration, both in number and repertoire, following intensive chemotherapy, critically depends on the age of the patient [16]. The incidence of invasive candidiasis is higher in the pediatric age group, with the highest risk in neonates [17, 18]. Candida infections in older children are more similar to those in adults. In children aged younger than 1 year, the incidence of *C. parapsilosis* is considerably higher than that of *C. glabrata*, whereas in adolescents, the incidence of *C. glabrata* may exceed the incidence of *C. parapsilosis* [19]. Overall, the rate of mortality due to invasive candidiasis is lower in children compared with adults [20].

Invasive aspergillosis, in contrast to invasive candidiasis, is rare in neonates, but occurs more frequently in older children. The overall fatality rate of invasive aspergillosis varies from 53 %, similar to that seen in adult patients, to as high as 70 % [5, 21] and it significantly contributes to the mortality of immunocompromised children. In a large national retrospective study in the USA, 18 % of children with invasive aspergillosis died in the hospital, compared to 1 % of similarly immunocompromised children without invasive aspergillosis [22].

Relatively little is known regarding Mucormycosis in children. A systematic literature review including reports back to 1939 identified a total of 157 reported children with Mucormycosis [23]. Whereas prematurity was the most common risk factor in pediatric patients, diabetes and underlying malignancy were seen in both children and adults developing invasive mucormycosis. Compared with children, the mortality of Mucormycosis appeared to be lower in adults, which might be due to a lower rate of dissemination [24].

## Diagnosis

The armamentarium available for diagnosing IFIs includes direct or indirect methods of detection [25]. No test is perfect and it is necessary to perform several diagnostic tests to achieve maximum accuracy [25]. Direct methods include the demonstration of fungal elements in blood or body fluids by microscopy and culture or from tissue by histopathology and culture of homogenized tissue. This can only be achieved by getting samples with invasive procedures from patients. In some cases, such as pulmonary aspergillosis, this is difficult to perform because of the risks associated with taking a lung biopsy in a sick child in whom contraindications for invasive procedures like thrombocytopenia may be present.

Culture allows the identification of types and species of the fungi and provides a means of testing susceptibility of the fungi to antifungal agents. However, culture is a time-consuming process which is a major limitation as it delays the onset of therapy. Moreover, the yield is suboptimal (about 50–60 %) in cases where there is fungemia [26]. The results of histology may come faster but may only identify fungi to a certain degree. This may help in starting direct initial therapy but ultimately culture is needed to properly define the fungi and conduct susceptibility tests [26].

Indirect methods were introduced to try to overcome some of the limitations of the direct methods, particularly to reduce the time of diagnosis. Due to the high morbidity and mortality, antifungal therapy is started empirically when there is a high suspicion of fungal infection. However, this leads to the exposure of patients who do not have IFIs to antifungal therapy and thus the risk of adverse reactions. Preemptive strategies where only patients considered very likely to have IFIs are identified and treated have now been adopted by most ICUs. These strategies are based on guidelines, which help identify these patients [27]. Indirect methods play a major role in these guidelines and include commercially available assays against antigens in the fungal cell wall to detect galactomannan (GM) or β-1,3-d-glucan (BDG) for detection of Aspergillus and most fungal species, respectively [28].

Other indirect tests available include detection of DNA sequences by polymerase chain reaction not only in blood but also especially in bronchoalveolar washings and other body fluids. GM is fairly specific for Aspergillus, but it may have cross reactivity with GM present in the cell wall of *Penicillium sp*. and other organisms. It has a very good sensitivity, which has been found to perform well in both children and adults in prospective studies. BDG is a component of the cell wall of many pathogenic fungi and did not perform as well in children as in adults [29]. These assays have been introduced into the Revised European Organization for Research Cancer Treatment Mycosis Study Group (EORTC/MSG). The test in combination with clinical (including radiological) findings allows one to classify the diagnosis of IFI as definite, probably, or possible. This classification is for clinical trials and not necessarily for diagnosis in the individual patient. The classification emphasizes the difficulty in diagnosis of IFIs [30].

The published data on specificity and sensitivity of diagnostic approaches such as the Aspergillus galactomannan (GM) test in the pediatric population are quite limited [31]. This is because many clinical trials enroll adult patients only, and because sub-analyses of pediatric data from larger trials enrolling both pediatric and adult patients, as well as prospective studies in children, are limited in their interpretability by small patient numbers. One study analyzed GM in 3294 serum samples from a total of 728 patients. The specificity in the entire study population was 94.8 %; however, it was significantly lower in the 42 children included in the study (47.6 %) [32]. In contrast to these findings, the specificity of the GM assay was 97.5 % in a prospective study in 64 children undergoing HSCT [33]. Similarly, the diagnostic sensitivity and specificity of β-d-glucan (BDG) for the diagnosis of candidiasis seem to be adequate in adult patients, whereas the value in the pediatric population is not clear at all. Notably, a recent study evaluated BDG levels in children specifically not at risk for IFI and reported higher baseline levels of the assay in children compared with adults [34].

Recently, a new indirect test was developed: T2MR and T2Candida, a miniaturized magnetic based diagnostic approach that measures how water molecules react in the presence of a magnetic field. The method is capable of detecting molecular targets such as DNA. It is reported to be able to detect Candida on whole blood in cases where the concentration of Candida in the blood is too low to be detected by blood culture as would occur in culture negative disseminated Candidiasis. Trials are still ongoing to determine the economic and medical impact of this new diagnostic tool [35].

## Imaging

### Anatomical imaging

In clinical practice, medical imaging and noninvasive testing such as GM, BDG, and nucleic acid techniques are all part of the diagnostic pathway to track fungal infections, particularly for invasive aspergillosis [36, 37]. Plain radiographs, ultrasound (US), conventional CT, HR CT, and MRI all play a role in the diagnosis and management of fungal infections [11, 37, 38], but all have their limitations. MRI is particularly useful for identifying infections in the central nervous system (CNS) and the facial sinuses, which can be rapidly fatal in acute sinusitis [12]. HR CT has been found valuable in settling the diagnosis of pulmonary IFIs. 70 % of IFIs are believed to involve the lungs in the immunocompromised patient. CT is not useful for acute sinusitis but useful in a chronic setting where it can evaluate changes in the bone. US, CT, and MRI are useful in diagnosing metastatic deposits of IFIs in the intra-abdominal viscera particularly the spleen, kidney, and liver. MRI, however, was unable to diagnose a spondylodiscitis due to an IFI in a series where it showed good accuracy for bacterial spondylodiscitis [39]. We will now discuss more thoroughly the two most used anatomical imaging modalities in patients with invasive fungal infections.

#### MRI in the central nervous system

**Fig. 1:**
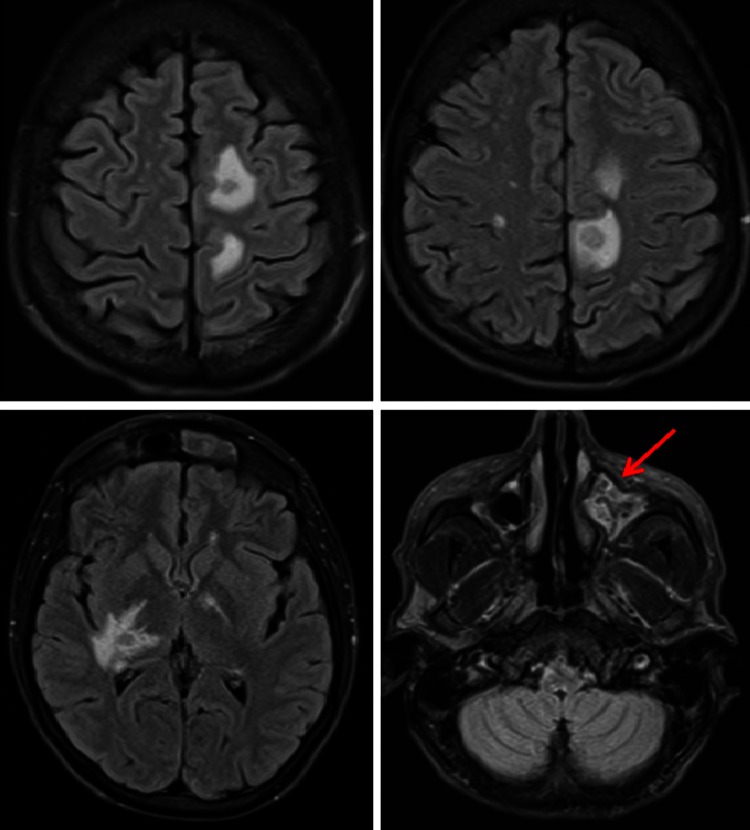
MRI scan of the brain in a patient with acute myeloid leukemia and CNS aspergillosis. It shows multiple ring enhancing lesions in the internal border zone bilaterally (border zone between lenticulostriate perforators and the deep penetrating cortical branches of the middle cerebral artery (MCA) or at the border zone of deep white matter branches of the MCA and the anterior cerebral artery. *Red arrow* shows thickening of the mucosa of the frontal sinus due to acute sinusitis

Early hematogenous spread of IFIs initially produces a cerebritis without abscess formation which cannot be easily detected by MRI. Later frank abscesses form that can be picked up by post-gadolinium MRI as reduced diffusion due to high viscosity and cellularity of fungal pus that may precede ring enhancement (Fig. 1). The reduced diffusion in contrast to pyogenic pus is usually heterogeneous. In disseminated IFIs, a mycotic vasculitis-mediated septic infarction occurs predominantly at the gray-white junction or perforating arterioles. This is seen as subtle enhancement and heterogeneous reduced diffusion on MRI. This anatomical distribution is different from other infarcts, cerebritis, or abscesses. Cryptococcus or Aspergillus may seed the cerebrospinal fluid giving variable appearance of enhanced or non-enhancing lesions of the meninges, choroid plexus, or ependyma. They may also produce hydrocephalus with or without white matter edema. In sinusitis, there is usually enhancement with reduced diffusion noted in the inferior frontal lobe (Fig. 1). There are specific signs for particular fungal infections beyond the scope of this review [40].

#### HR CT for pulmonary aspergillosis

**Fig. 2:**
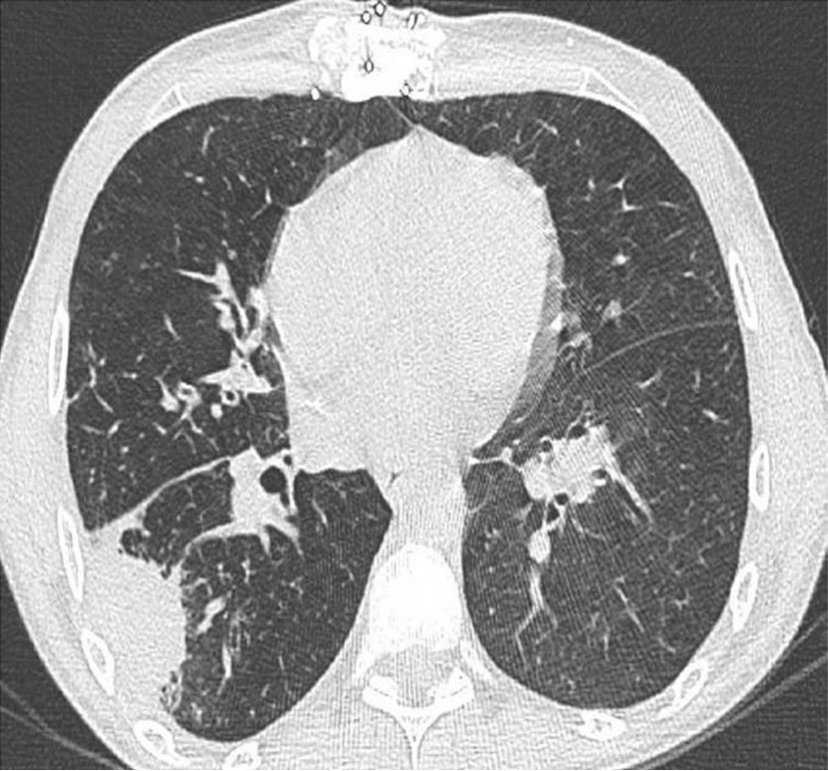
HR CT chest scan demonstrating a biopsy-proven *Aspergillus sp.* infection. The pleural-based lesion shows surrounding glass ground appearance on the free edge. The presence of this intrapleural lesion shows the halo sign, a lesion typically seen early in Aspergillosis

The introduction of HR CT has allowed earlier preemptive therapy of many patients by identifying lesions highly suggestive of IFIs in the presence of a positive indirect test. This is particularly true for invasive pulmonary aspergillosis. Spores of *Aspergillus sp.* usually enter the body through sinuses or respiratory tract infecting them. Aspergillus infects airways resulting in bronchopneumonia in the early stages, which may be normal on chest radiograph. As the disease progresses nodular appearance or patchy consolidations may appear. Aspergillus frequently appears as a single or multiple area of rounded consolidation, which may cavitate. In adults, two key signs exist on HRCT suggestive for invasive pulmonary aspergillosis: the halo sign and the air crescent sign. The halo sign is a ground glass opacity surrounding a pulmonary nodule or mass and represents hemorrhage (Fig. 2). This sign appears transiently in the disease and soon the finding changes to nonspecific findings. The air crescent sign describes the crescent of air that can be seen in invasive aspergillosis. Both the halo sign and the air crescent sign are common and highly suggestive for invasive mold infection in adult patients [30]. However, various retrospective studies demonstrated that these CT findings are less specific in children. In children, other findings including segmental and multilobar consolidation, peripheral infiltrates, multiple small nodules, and larger peripheral nodular masses are common, whereas the halo sign is rarely present [41–44]. The use of HRCT in pulmonary candidiasis is less obvious. Pulmonary candidiasis usually gives small nodular lesions, which do not cavitate [11, 45, 46]. In general, the findings of IFIs in children on HR CT are not specific and may occur in other conditions like bacterial infections or malignancies [10].

### Molecular imaging techniques and hybrid imaging

Nuclear medicine techniques such as positron emission tomography (PET) detect in vivo pathophysiological changes before anatomical changes are observed [47, 48]. Modern anatomical imaging modalities such as CT and MRI depend on structural resolution for visualizing disease. They are generally of limited value in detecting early disease irrespective of the cause. Functional and metabolic images are needed to complement their role in diagnosis of infection. Modern hybrid imaging modalities (PET/CT and PET/MRI) provide a unique opportunity to combine the excellent anatomical resolution with metabolic information to diagnose, localize, and stage IFIs at a very early stage [49]. PET/CT has the advantage of being a whole-body imaging technique; it is not limited to only one region of the body, so it can provide information of the whole body in one imaging session and thus is likely to pick up infectious foci which may not yet have become clinically apparent.

### ^18^F-fluorodeoxyglucose PET

The most commonly used tracer in molecular imaging of IFIs is ^18^F-fluorodeoxyglucose (FDG). We performed a literature search about the role of FDG-PET in IFIs (adults and children) by entering the words FDG and invasive fungal infections, FDG and candidiasis, FDG and aspergillosis, FDG and molds, and FDG and all other existing fungi. The references of these articles were also screened and relevant articles were also included. All included papers have been summarized in 2 tables; the first one (Table 1) provides an overview of articles that showed the role of FDG-PET in IFIs in the lung, which accounts for 70 % of IFI cases. Table 2 shows the extrapulmonary involvement of IFIs, grouped by the site of the body where the IFI occurred. FDG-PET showed avid uptake across a wide range of IFIs in different sites of the body. In the following paragraphs, we provide an overview of what FDG-PET offers in imaging IFIs in both adults and children.

**Table 1:** Overview of available papers in literature on pulmonary IFIs

Author and year	Journal	Type of fungal infection	Number of patients	Significant additional findings with FDG-PET
Camus et al. [67]	Anticancer Res	Candidiasis	3	Useful in evaluation of febrile neutropenia
		Aspergillosis	4	
Kono et al. [53]	Clin Nucl Med	Pneumocystis jirovecii	1	Positive when CT was equivocal
Reyes et al. [58]	Lung	Coccidiomycosis	12	SUV cannot differentiate between fungal and malignant lesions; beware of false positive findings in patients with lung cancer
Sharma et al. [59]	AJR Am J Roentgenol	Aspergillus	1	Useful in assessing if IFI deposit was active
		Cryptococcus	1	Mimics lung cancer
		Mucormycosis	1	Rare presentation detected by PET
Wang et al. [59]	Int J Infect Dis.	Cryptococcus	1	Mimics primary lung cancer with bone metastasis
Kim et al. [74]	J Comput Assist Tomogr	Aspergillosis	24	Distinguished invasive aspergillosis from noninvasive pulmonary aspergillosis
Hamerschlak et al. [61]	Einstein (Sao Paulo)	Cryptococcus	1	False positive lymphoma
Baxter et al. [72]	Thorax	Aspergillosis	1	Mimics lung cancer
Ahn et al. [71]	Thyroid	Aspergillosis	1	Mimics metastatic thyroid cancer
Vahid et al. [54]	MedGenMed	Blastomycosis	1	Mimics lung cancer
Nishikawa et al. [70]	Kyobu Geka	Aspergillosis	1	Mimics a tuberculoma
Hot et al. [50]	Clin Microbiol Infect	Aspergillosis	9	Detected all lesions seen by HRCT
		Zygomycosis	2	
		Histoplasmosis	2	
		Coccidioidomycosis	1	
Dang et al. [55]	Clin Nucl Med	Mucormycosis	1	Rare presentation of IFI detected by PET guided biopsy
Xu et al. [73]	Clin Nucl Med	Candidiasis	1	Monitor antifungal therapy
Nakazato et al. [79]	Ann Hematol	Pneumocystis jirovecii	1	Useful for early diagnosis of IFI
Avet et al. [52]	EJNMMI	Candidiasis	1	Lesion detected on completion of antifungal. Not previously detected
Sonet et al. [69]	Ann Hematol	Aspergillosis	1	Mimics lymphoma
Vahid et al. [54]	MedGenMed	Blastomycosis	1	Mimics primary lung cancer
Igai et al. [62]	Eur J Cardiothorac Surg	Cryptococcus	6	Mimics lung cancer
Salhab et al. [63]	J Cardiothorac Surg	Histoplasmosis	1	Mimics primary lung cancer
Bleeker-Rovers et al. [75]	J Nucl Med	Candidiasis	9	Useful in detecting metastatic foci of IFI
Bleeker-Rovers et al. [51]	Clin Microbiol Infect	Candidiasis	3	Lung lesions were not seen on CT
Wilkinson et al. [68]	Clin Nucl Med	Aspergillosis	1	Mimics lung cancer
Theobald et al. [66]	Radiologe	Aspergillosis	2	Determined extent of spread of disease
Croft et al. [60]	Lung Cancer	Histoplasmosis	2	False positive in lung cancer evaluation
		Coccidiomycosis	1	
Franzius et al. [65]	Clin Nucl Med	Aspergillosis	1	Useful for monitoring therapy
Ozsahin et al. [64]	Blood	Aspergillosis	1	Monitored infection to allow during immune suppressive procedure
O’Doherty et al. [56]	J Nucl Med	Cryptococcus	2	Helped determine cause of symptoms in HIV patients
Chamilos et al. [94]	Med Mycol	Aspergillosis	8	Detected all lesions seen on other imaging modalities, Monitored therapy and revealed extra pulmonary occult site
Chamilos et al. [94]	Med Mycol	Zygomycosis	5	Detected all lesions seen on other imaging modalities, was helpful in distinguishing infection from malignancy
Ritz et al. [95]	Eur J Pediatr	Zygomycosis	1	Therapy monitoring
Ho et al. [96]	Br J Haematol	Aspergillosis	1	Therapy monitoring
Go et al. [97]	Acta Neurochir	Aspergillosis	1	Mimics lung cancer
Eubank et al. [98]	J Clin Oncology	Aspergillosis	1	Mimics lung cancer

**Table 2:** Overview of available papers in literature on extrapulmonary IFIs by site of infection

Site	Author date	Journal	Type of IFI	No of patients	Relevant comment or finding of FDG-PET
Liver	Hot et al. [50]	Clin Microbiol Infect	Aspergillus	1	3 liver lesions noted
Liver			Candida	10	More lesion found by FDG-PET in the liver (3 cases). CT and US did not see lesion in one case
Liver	Miyazaki et al. [80]	Ann Hematol	Yeast-like	1	Useful for therapy monitoring
Liver	Xu et al. [73]	Clin Nucl Med	Candida	3	Useful for therapy monitoring
Liver	Teyton et al. [85]	Clin Nucl Med	Candida		Useful for therapy monitoring
Liver	Avet et al. [52]	EJNMMI	Candida	1	Detected after completion of antifungal therapy
Liver	Sharma et al. [49]	AJR Am J Roentgenol	Candida	1	FDG-PET found more lesions than CT
Spleen	Hot et al. [50]	Clin Microbiol Infect	Candida	7	FDG-PET found more lesions in the spleen in 3 cases
Spleen	Tibúrcio et al. [76]	BMC Pediatr	Candida	1	FDG-PET Helps in showing IFI metastatic foci when other modalities were equivocal
Spleen	Avet et al. [52]	EJNMMI	Candida	1	Detected active lesion after completion of antifungal therapy
Spleen	Teyton et al. [85]	Clin Nucl Med	Candida	1	Useful for therapy monitoring
Spleen	Ritz et al. [95]	Eur J Pediatr	Zygomycosis	1	Detection of occult (extra pulmonary) lesion and therapy monitoring
Kidneys	Avet et al. [52]	EJNMI	Candida	1	Detected active lesion after completion of antifungal chemotherapy
Bones	Sharma et al. [49]	Sharma P et al. 2014	Cryptococcus	1	FDG-PET shows systemic IFIs involving bone
			Mucormycosis		
Bone	Hot et al. [50]	Clin Microbiol Infect	Mycetoma	2	FDG-PET demonstrates soft tissue and bone involvement
Joints			Phomopsis	1	
Bone	Wang et al. [59]	Int J Infect Dis	Cryptococcus		Mimics metastatic cancer-primary in lung
Bone	Karunanithi et al. [78]	Clin Nucl Med	Histoplasma	1	Useful for rare presentation of IFIs- isolated sternum
Bone	Morooka et al. [83]	Jpn J Radiol	Candida	1	Directed biopsy to diagnose IFI
Joints	Fuster D et al. [46]	EJNMMI	Aspergillus	1	Diagnosed IFI spondylodiscitis when MRI did not
Adrenal gland	Altinmakas et al. [77]	Clin Imaging	Candida	1	1. Alerts the possibility of IFIs when intense bilateral adrenal uptake is observed 2. Therapy monitoring for Refs. [88, 89] in addition to above
			Histoplasma	1	
	Sharma et al. [49]	AJR Am J Roentgenol	Cryptococcus	1	
			Histoplasma	1	
	Padma et al. [87]	Indian J Med Res	Histoplasma	1	
	Kasaliwal et al. [88]	Clin Nucl Med	Histoplasma	1	
	Tsai et al. [89]	Clin Imaging	Histoplasma	1	
	Umoeka et al. [90]	Eur Radiol	Histoplasma	1	
CNS	Hanson MW et al. [86]	J Comput Assist Tomogr	Aspergilloma	1	FDG-PET useful for guiding biopsy
CNS	Dubbioso et al. [92]	J Neurol Sci	Cryptococcus	1	Useful for therapy monitoring Rare CNS presentation
CNS	Chamilos et al. [94]	Med Mycol	Aspergillus	1	Revealed an occult infection- whole-body imaging done
CNS	Hanson et al. [100]	J Comput Assist Tomogr	Aspergillus	1	Detected CNS involvement
Kidney	Sharma et al. [49]	AJR Am J Roentgenol	Mucormycosis	1	Defined extent of sinusitis and identified involvement of distant organs
Urinary bladder				1	
Maxillary sinuses with nasopharynx and bone extension				1	
Hypopharynx			Histoplasma	1	Lesion clearly delineated and distant spread (adrenal)
Frontal and ethmoidal sinuses	Altini et al. [12]	Clin Nucl Med	Mucormycosis	1	Correctly predicted disease progression in contrast to MRI Treatment monitored with FDG
Ethnoidal sinus with extension to the nasopharynx and nasal cavity	Liu et al. [57]	Clin Nucl Med	Mucormycosis	1	Serial scans helped modify antifungal therapy.
Maxillary sinus	Kawabe et al. [99]	Ann Nucl Med	Aspergillus	1	Compared to ^67^Ga citrate uptake
Aortic valve (prosthetic)	Wallner et al. [82]	Herz	Candida	1	Useful for evaluation of therapy for IFI endocarditis
Aorta	Roux et al. [81]	Rev Med Interne	Candida	1	Contributed to the diagnosis of mycotic aneurysm
Lymph nodes	Sharma et al. [49]	AJR Am J Roentgenol	Cryptococcus	2	Mimics malignant metastatic node
Lymph nodes	Nakazato et al. [79]	Ann Hematol	Pneumocystis jirovecii	1	Useful for early diagnosis of IFI
Lymph nodes	Mackie et al. [91]	Clin Nucl Med	Histoplasma	1	Mimics malignant metastatic node
Muscles and myocardium	Avet et al. [52]	EJNMMI	Candida	1	Lesions previously undetected were identified on completion of antifungal therapy
Esophagus	Shrikanthan et al. [84]	Clin Nucl Med.	Candida	1	Uptake concealed esophageal cancer

#### Value of FDG-PET/CT in IFIs

The most compelling evidence for the use of FDG-PET/CT in IFIs is from a prospective study involving a wide range of fungi in 30 consecutive adults and children with probable or proven IFI [50]. FDG-PET showed uptake in all areas noted by conventional imaging making it at least as sensitive as the total of all other imaging studies performed, including MRI, CT, and US. Furthermore, in this study, FDG-PET detected more lesions in the liver and spleen in some cases of hepatosplenic candidiasis. This was in support with earlier reports which also noted invasive candidiasis lesions which had not been detected on conventional imaging [51, 52]. These metastatic foci most likely were identified early in disease where the anatomical changes associated with infection were not visible yet. In patients with aspergillosis where HR CT has made an impact of early diagnosis, FDG-PET/CT not only detected all active lesions, but also was able to correctly distinguish inactive noninvasive aspergilloma from active disease. This is of particular importance in children in whom HSCT, SOT, or chemotherapy is being considered. This study further highlights the role of FDG-PET/CT in therapy response, which was assessed in 20 % (6 out of 30) of their patients. Due to the small number of patients that were scanned also for therapy response, they could not conclude if FDG-PET is also useful for therapy evaluation [50].

#### Role of FDG-PET in staging IFIs

**Fig. 3:**
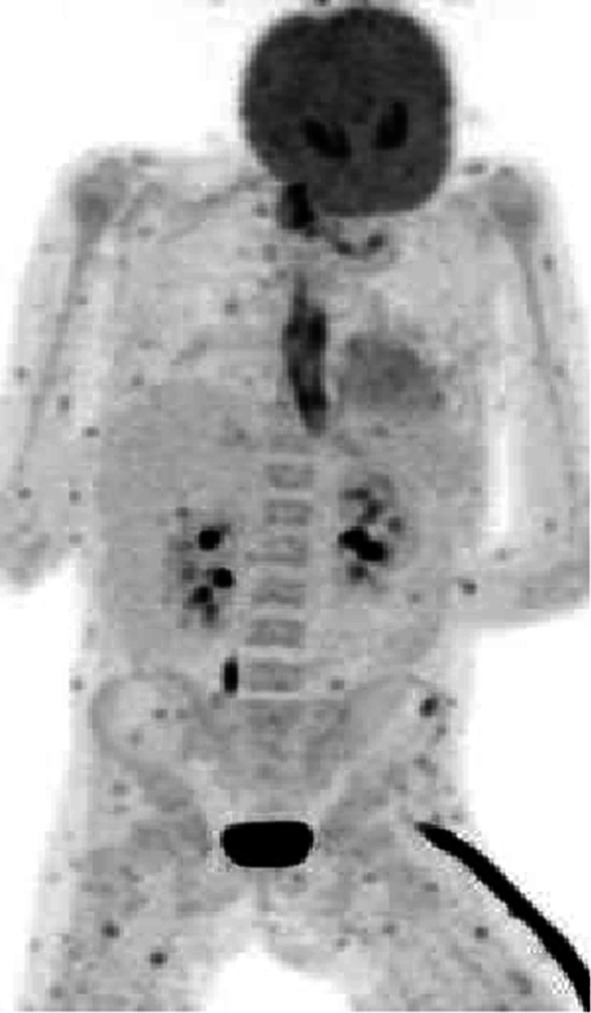
Disseminated candidiasis in a 10-year-old girl with acute lymphocytic leukemia on chemotherapy. The pattern of widespread lesions in the muscles and involvement of the esophagus points towards an infection with candida (later on proven by biopsy)

The overall agreement of all studies is that FDG-PET/CT is useful in staging IFIs. It has the advantage of being a whole-body imaging modality and is able to detect metastatic infectious foci, which are not detected by other imaging studies. This phenomenon was consistently demonstrated in a number of papers [49–51]. It will be helpful before the onset of therapy to know the extent of the infection and the organs involved, not only to correctly stage it during infection, but also to decide later if the infection disappeared and after completion to exclude recurrence of the fungal infection. An example of a patient (10-year-old girl) with disseminated fungal infection is shown in Fig. 3.

**Fig. 4:**
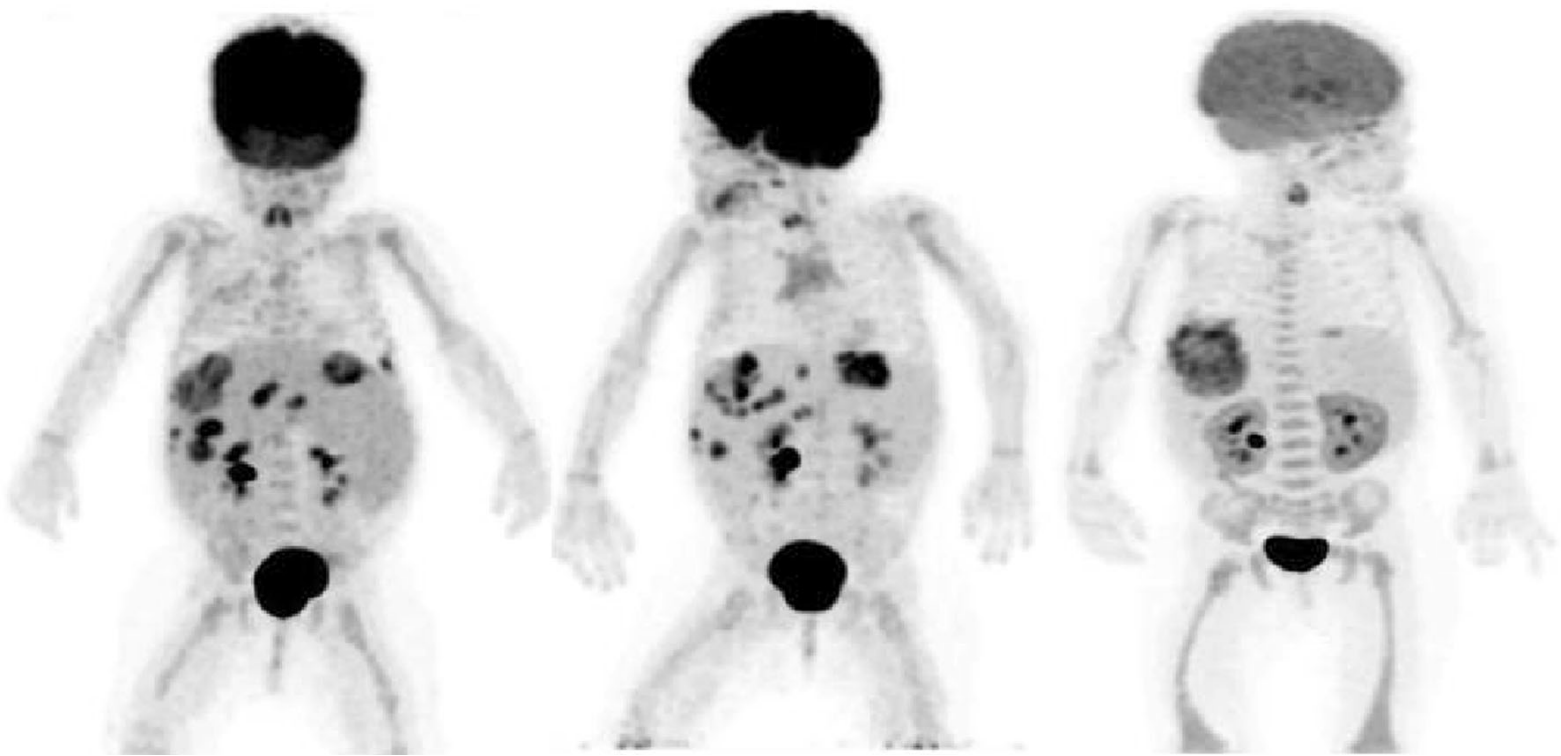
Example of use of FDG-PET in therapy monitoring in a 2-year-old girl with Langerhans cell histiocytosis and bone marrow transplantation. She was diagnosed (after biopsy) with aspergillus lesions in the liver. **a** Baseline FDG-PET scan, MIP image, revealing multiple fungal lesions in the liver. **b** FDG-PET scan after 6 months of antifungal therapy, showing decrease in uptake of some liver lesions, but increase of other liver lesions. Based on these findings, antifungal treatment was switched. **c** FDG-PET scan 3 months after therapy switch, revealing disappearing of all liver lesions expect one which became larger in time. Eventually this lesion was surgically removed, showing an encapsulated fungal lesion, which could not be reached by the antifungal drugs. Note also the decreased uptake in the brain at the third scan. This scan was performed under sedation

Despite the aspecific uptake of FDG, a possible diagnosis can be made based on the uptake pattern of FDG and in light of the clinical findings, and other diagnostic tests. However, histological confirmation must always be performed for a final diagnosis. FDG-PET is able to define the site(s) of active infection where biopsy is likely to provide the correct diagnosis. The finding of high bilateral uptake in the adrenal glands in an immunocompromised patient must raise the suspicion of a fungal infection. The presence of multiple round lesions widely spread throughout the body or in the liver or spleen in a patient with risk factors for IFIs should lead to suspect Candida infection. The predictive value of this diagnosis is further strengthened if there is also esophageal uptake to suggest esophageal candidiasis. *Aspergillus sp.* lesions are usually bigger and may show a central area of decreased metabolism (cold center) most likely to the angio-invasive nature of the fungi causing necrosis due to an infective thrombotic vasculitis (see also Fig. 4).

#### Role of FDG-PET in therapy monitoring of patients with IFIs

Several authors demonstrated the ability of FDG-PET in monitoring therapy of patients with IFIs [12, 52, 64, 65, 73, 80, 82, 84, 85, 92]. This is particularly crucial in disseminated and deep organ infection without fungemia. Some treatment protocols recommend that antifungal drugs should be given for a number of weeks in patients with suspected IFIs, also when blood cultures are negative. IFIs in immunocompromised patients are life threatening, and antifungal therapy is not only extensive, but must also be prolonged for a long time (depending on which fungal infection 6 months–2 years). In cases where there is no fungemia, the duration of therapy becomes ambiguous. In case of immunocompromised patients being evaluated for HSCT or SOT, it is important to know if all residual IFI are cleared before these procedures are undertaken. Clearly, it is of invaluable importance to have a noninvasive whole-body technique to localize all fungal lesions and to have a possibility to monitor disease activity to decide if therapy can be stopped, or should be prolonged or switched. In disseminated candidiasis, lesions seen on US, CT, or MRI have been found to persist for a long time after successful antifungal treatment due to, e.g., fibrosis and therefore these imaging modalities may have limited roles in assessing therapeutic effect and guiding therapy. FDG-PET will provide accurate information on therapeutic response especially for residual focal deposits in disseminated candidiasis. In one publication, FDG correctly predicted disease progression where MRI findings suggested improvement [12]. In another study, after completion of antifungal therapy for hepatosplenic and renal abscess before restarting chemotherapy, FDG-PET/CT detected lesions in the skeletal, cardiac muscles and the lungs showing antifungal treatment failure; hence, a different antifungal was given and lesions resolved [52].

#### FDG-PET in IFIs in children

In our review of the available literature, we found that data on the use of FDG-PET in children were very scarce. 15.3 % of the available papers (8/52) included children (Table 3), but even within these papers children were under represented with only 24.3 % (9/37) of cases reported involving patients less than 18 years. In the children reviewed, most were infected by the two most common causes of IFIs, *Aspergillus* and *Candida sp*. There were more cases involving *Aspergillus* than *Candida sp.* (5 vs. 2), probably underscoring the higher mortality associated with the former. There were two cases of IFIs caused by one of the rarer causes of IFI, Zygomycosis (which includes Mucormycosis). In the limited number of cases presented, the findings on FDG-PET did not differ significantly between children and adults. Indeed, in a 6-year-old patient with IFI, it was the pattern of uptake that was similar to the uptake in adults. This enabled a diagnosis of a Candida infection to be made rather than a recurrent malignant disease when other imaging modalities were unhelpful in this regard [76]. In all cases of children presented, the IFI lesions showed uptake similar to the cases in adults. The response of FDG-PET to antifungal therapy was also similar. In one case, in an era where there were limited options of antifungal therapy compared to the current situation, FDG-PET was able to carefully monitor the IFI in a patient which had not responded to antifungal therapy and allowed bone marrow transplantation to be carried out [44]. The differences in imaging findings between children and adults with aspergillosis on HR CT had to do with cavitations which occur later in the disease. As FDG-PET relies on molecular changes which precede these anatomical changes, it is unlikely this difference noted in HR CT would be observed with FDG-PET. FDG-PET, as is the case in adults, was able to detect lesions in children with neutropenia [56]; this is consistent with a review of FDG-PET in febrile neutropenia [93]. In one case involving *Mucormycosis sp.* in children, FDG-PET was not only able to detect lesions but also proved to be superior in monitoring the disease when compared to MRI [12].

**Table 3:** Overview of available literature studies where children with IFIs were documented

References	Journal	Age	Underlying condition	Fungi, site of IFI, value of ^18^F-FDG
Altini et al. [12]	Clin Nucl Med	16 years	Acute myeloid leukemia and completed chemotherapy 3 months	*Mucormycosis sp.*-nasal sinuses, valuable tool for initial staging compared to MRI and useful for therapy monitoring
Hot et al. [50]	Clin Microbiol Infect	6 years	Chronic granulomatous disease and interferon-gamma therapy	*Aspergillus sp.*-lung, uptake in a suspected case of invasive pulmonary was persistent on follow-up
Avet et al. [52]	EJNMMI	16 years	Acute leukemia and completion of chemotherapy	*Candida sp*.-disseminated. Previously undiagnosed metastatic foci detected after completion of antifungal therapy
Tibúrcio et al. [76]	BMC Pediatr	6 years	Neuroblastoma on treatment with febrile neutropenia	*Candida sp*. –spleen, helped diagnosis, other imaging modalities were equivocal
Franzius et al. [65]	Clin Nucl Med	3 months (2 pts-twins)	Chronic granulomatous disease	*Aspergillus sp.*-lung, Staged disease, diagnosed in twin sister and monitored therapy in both
Ozsahin et al. [64]	Blood	8 years	X-linked chronic granulomatous disease	*Aspergillosis sp*.- lungs, accurate monitoring of infection during an immunosuppressive procedure
Theobald et al. [66]	Radiologe	>8-week old premature infant	Chronic granulomatous disease	*Aspergillus sp.*-lung. Diagnosed and stage disease in an asymptomatic twin
Ritz et al. [95]	Eur J Pediatr	9-year old	Burkitts lymphoma on chemotherapy	*Zygomycosis sp.*-lung and spleen. Detected occult infection and used to monitor therapy

Figure 4 shows the use of FDG-PET in clinical practice and how FDG-PET is important even with the development of several new diagnostic tests that are more sensitive than blood culture such as T2MR and T2Candida. These tests provide the clinician an idea of the presence of the fungi even at very low levels, but are not able to tell how the fungi in different lesions in the body respond to the antifungal therapy. As this figure clearly demonstrates, there was response of some liver lesions but also new lesions developed after 6 months of antifungal therapy. Based on this second FDG-PET scan in this 3-year-old girl, a new antifungal drug regimen was started. Three months after the start of this new treatment, most liver lesions responded completely leaving only one large lesion with a large necrotic center and increased peripheral metabolic activity. Eventually, this lesion was surgically removed and pathology indeed revealed an encapsulated fungal lesion. This different response of the different lesions illustrates how FDG-PET imaging is able to help in therapy decision making in children with IFIs.

#### Limitations of FDG-PET to image IFIs in children

First of all, as mentioned earlier, the nonspecific uptake of FDG makes it impossible with this tracer to differentiate completely between fungal infections, bacterial infections, malignancy, or inflammatory lesions. Furthermore, FDG is taken up in high amounts in the brain and in the heart and excreted by the kidneys and the bladder, thereby limiting the detection of fungal infections in these organs. In some of the reported cases, FDG-PET was used to detect IFIs in the brain [91]. In our opinion, MRI should be used when having suspicion of fungal lesions in the brain. Hybrid imaging with MRI will most likely overcome this limitation [101]. Physiological FDG uptake in the heart can be avoided by using a low carbohydrate diet for 24 h before the administration of the FDG, thereby forcing the heart to switch from a glucose metabolism to a free fatty metabolism. However, this preparation may not always be possible to execute in very sick children on the ICU. Fungal lesions in the kidneys can be visible on FDG-PET/CT, by clearly defining which uptake is based on fungal lesions in the parenchyma and which uptake is caused by excretion by the collecting system.

Overall, the FDG-PET/CT procedure (depending if a low dose CT or also a diagnostic CT is performed) takes approximately 80–100 min, including the 60 min waiting time between administration of FDG and start of the scan. This may be too long for children, especially since they are not allowed to move or speak. Therefore, in some cases, sedation may be required. In all cases, both the child and the parents or guardian should be fully aware of the procedure and given a familiarization tour. This may reduce anxiety and obviate the need for sedation. Recently, educative cartoon books for children were published that explain the imaging procedure in a funny easy way to children [102]. This may also be helpful to reduce anxiety before the procedure.

A last limitation: both PET and CT are procedures involve the use of ionizing radiation. The tissues of children are particularly sensitive to radiation. Both the referring clinicians and the nuclear medicine physicians have to be fully aware of this. In situations where several PET/CT scans are performed for therapy follow-up, we recommend to perform a diagnostic CT for the initial PET/CT study and follow-up with only a low dose CT for anatomical localization and attenuation correction. Though not widely available at this moment, PET/MRI has the advantage of the non-radiation part of the MRI and could be of absolute use in children who require several follow-up scans.

### Other available tracers

Fungal infections have been imaged in the past with several SPECT tracers such as technetium-99 m (^99m^Tc)-labeled fluconazole, ^99m^Tc-labeled ubiquicidin, and ^99m^Tc-labeled lactoferrin-derived peptides, such as ^99m^Tc-CBP21 and T^99m^Tc-hLF 1-11. The peptides were rapidly taken up at sites of infection and not at inflammatory but sterile sites; however, they did not discriminate between fungi and bacteria. ^99m^Tc-fluconazole accumulated only in viable Candida infection and uptake correlated very well with the number of fungi present. It did not accumulate in bacteria of *Aspergillus fumigates.* All these SPECT-based tracers also showed promises for therapy monitoring [103]. Fluconazole was also labeled with PET radionuclide ^18^F. However, the results were relatively disappointing. There was a poor accumulation at infected sites and high amount of activity was detected in the liver decreasing its sensitivity to detect *Candida* infections. The reason of the difference between this PET tracer and its SPECT equivalent was the different labeling methods resulting in ^18^F-fluconazole being much more lipophilic than ^99m^Tc-fluconazole, resulting in poorer imaging characteristics [103, 104].

^68^Ga-labeled tracers have recently attracted great clinical interest for molecular imaging procedures using PET. In mice ^68^Ga-citrate labeled with Triacetylfusarinine C (TAFC) and ferrioxamine E (FOXE) has been shown to be highly sensitive for Aspergillus imaging [105]. TAFC and FOXE are common trihydroxamate-type siderophores with relatively low molecular weight produced by fungi, bacteria, and some plants for scavenging iron making it available to the organism. These ^68^Ga-siderophores were found to be superior to ^68^Ga–citrate which had a slow excretion and high blood pool uptake [105, 106]. ^67^Ga-citrate was previously used in fungal infections and in one study it was compared with FDG [99]. It was noted to be able to accumulate in IFI lesions without blood vessel in an area where FDG showed minimal uptake; however, in areas of IFI with relatively preserved vasculature, uptake of FDG was more intense [99]. Further studies are necessary to determine whether ^68^Ga-citrate has advantages over FDG in humans. In another infection study, ^68^Ga citrate was found to detect with high sensitivity bacterial bone infections; no uptake was seen in sterile inflammation [107].

Newer radiolabelled targets such as chitin (a component of fungal cell wall that are absent from mammals) may be an interesting target for fungal infection imaging in the future. SPECT tracers targeted against chitin and the enzyme that degrades it (Iodine-123 labeled chitinase) has been found useful in imaging IFIs [103, 108, 109]. Further studies exploring novel radioligands capable of differentiating fungal from bacterial infections and also able to discriminate sterile inflammatory sites from infections are definitely warranted [103].

### Conclusions

Invasive fungal infections in children have a high morbidity and mortality. Early diagnosis and initiation of therapy is essential. Several diagnostic tests exist; however, all have their limitation. Literature on the use of nuclear imaging techniques in children with invasive fungal infections is scarce. However, based on studies in adults and based on clinical expertise, FDG-PET/CT offers important added value in both staging and therapy monitoring. FDG-PET is able to non-invasively detect all fungal lesions within the body (even in the presence of severe neutropenia [110]), is able to differentiate between active and inactive fungal infections, and is able to tell the clinician whether the antifungal therapy is helping, and should be stopped, switched, or continued.

Prospective multicenter studies should be started to achieve robust evidence based data to enable us to develop relevant diagnostic and therapeutic flow charts when to use which imaging modality in which stage of the disease.

## References

[1] Dornbusch HJ, Manzoni P, Roilides E, Walsh TJ, Groll AH (2009). Invasive fungal infections in children. Pediatr Infect Dis J.

[2] Ozsevik SN, Sensoy G, Karli A, Albayrak C, Dagdemir A, Belet N (2015). Invasive fungal infections in children with hematologic and malignant diseases. J Pediatr Hematol Oncol.

[3] De Pascale G, Tumbarello M (2015). Fungal infections in the ICU: advances in treatment and diagnosis. Curr Opin Crit Care.

[4] Brissaud O, Guichoux J, Harambat J, Tandonnet O, Zaoutis T (2012). Invasive fungal disease in PICU: epidemiology and risk factors. Ann Intensive Care.

[5] Steinbach WJ (2010). Epidemiology of invasive fungal infections in neonates and children. Clin Microbiol Infect.

[6] Posfay-Barbe KM, Zerr DM, Pittet D (2008). Infection control in paediatrics. Lancet Infect Dis.

[7] Blyth CC, Palasanthiran P, O’Brien TA (2007). Antifungal therapy in children with invasive fungal infections: a systematic review. Pediatrics.

[8] Kullberg BJ, Arendrup MC (2015). Invasive Candidiasis. N Engl J Med.

[9] Rammaert B, Desjardins A, Lortholary O (2012). New insights into hepatosplenic candidosis, a manifestation of chronic disseminated candidiasis. Mycoses.

[10] Kosmidis C, Denning DW (2015). Republished: the clinical spectrum of pulmonary aspergillosis. Postgrad Med J.

[11] Ahmad Sarji S, Wan Abdullah W, Wastie M (2006). Imaging features of fungal infection in immuno-suppressed patients in a local ward outbreak. Biomed Imaging Interv J.

[12] Altini C, Niccoli Asabella A, Ferrari C, Rubini D, Dicuonzo F, Rubini G (2015). (18)F-FDG PET/CT contribution to diagnosis and treatment response of rhino-orbital-cerebral mucormycosis. Hell J Nucl Med.

[13] Lehrnbecher T, Groll AH (2011). Invasive fungal infections in the pediatric population. Expert Rev Anti Infect Ther.

[14] Levy O, Martin S, Eichenwald Ganz T, Valore E, Carroll SF, Lee K, Goldmann D, Thorne GM (1999). Impaired innate immunity in the newborn: newborn neutrophils are deficient in bactericidal/permeability-increasing protein. Pediatrics.

[15] Yossuck P, Nightengale BJ, Fortney JE, Gibson LF (2008). Effect of morphine sulfate on neonatal neutrophil chemotaxis. Clin J Pain.

[16] Lehrnbecher T, Foster C, Vazquez N, Mackall CL, Chanock SJ (1997). Therapy-induced alterations in host defense in children receiving chemotherapy. J Ped Hematol. Oncol.

[17] Zaoutis TE, Argon J, Chu J, Berlin JA, Walsh TJ, Feudtner C (2005). The epidemiology and attributable outcomes of candidemia in adults and children hospitalized in the USA: a propensity analysis. Clin Infect Dis.

[18] Zaoutis T, Walsh TJ. Antifungal therapy for neonatal candidiasis (2007) Curr Opin Infect Dis 20(6):592–7 10.1097/QCO.0b013e3282f1bec9 17975409

[19] Malani PN, Bradley SF, Little RS, Kauffman CA (2001). Trends in species causing fungaemia in a tertiary care medical centre over 12 years. Mycoses.

[20] Pappas PG, Rex JH, Lee J, Hamill RJ, Larsen RA, Powderly W (2003). A prospective observational study of candidemia: epidemiology, therapy, and influences on mortality in hospitalized adult and pediatric patients. Clin Infect Dis.

[21] Burgos A, Zaoutis TE, Dvorak C, Hoffman JA, Knapp KM, Nania JJ, Prasad P, Steinbach WJ (2008). Pediatric invasive aspergillosis: a multicenter retrospective analysis of 139 contemporary cases. Pediatrics.

[22] Zaoutis TE, Heydon K, Chu JH, Walsh TJ, Steinbach WJ (2006). Epidemiology, outcomes, and costs of invasive aspergillosis in immunocompromised children in the USA, 2000. Pediatrics.

[23] Zaoutis TE, Roilides E, Chiou CC, Buchanan WL, Knudsen TA, Sarkisova TA (2007). Zygomycosis in children: a systematic review and analysis of reported cases. Pediatr Infect Dis.

[24] Roden MM, Zaoutis TE, Buchanan WL, Knudsen TA, Sarkisova TA, Schaufele RL (2005). Epidemiology and outcome of zygomycosis: a review of 929 reported cases. Clin Infect Dis.

[25] Miceli MH, Maertens J (2015). Role of non-culture-based tests, with an emphasis on galactomannan testing for the diagnosis of invasive Aspergillosis. Semin Respir Crit Care Med.

[26] Dekio F, Bhatti TR, Zhang SX, Sullivan KV (2015). Positive impact of fungal histopathology on immunocompromised pediatric patients with histology-proven invasive fungal infection. Am J Clin Pathol.

[27] Paramythiotou E, Frantzeskaki F, Flevari A, Armaganidis A, Dimopoulos G (2014). Invasive fungal infections in the ICU: how to approach, how to treat. Molecules.

[28] Ramos-Martín V, O’Connor O, Hope W (2015). Clinical pharmacology of antifungal agents in pediatrics: children are not small adults. Curr Opin Pharmacol.

[29] Koltze A, Rath P, Schöning S, Steinmann J, Wichelhaus TA, Bader P, Bochennek K, Lehrnbecher T (2015). β-D-Glucan screening for detection of invasive fungal disease in children undergoing allogeneic hematopoietic stem cell transplantation. J Clin Microbiol.

[30] De Pauw B, Walsh TJ, Donnelly JP (2008). Revised definitions of invasive fungal disease from the European Organization for Research and Treatment of Cancer/Invasive Fungal Infections Cooperative Group and the National Institute of Allergy and Infectious Diseases Mycoses Study Group (EORTC/MSG) Consensus Group. Clin Infect Dis.

[31] Bochennek K, Abolmaali N, Wittekindt B, Schwabe D, Klingebiel T, Lehrnbecher T (2006). Diagnostic approaches for immunocompromised paediatric patients with pulmonary infiltrates. Clin Microbiol Infect.

[32] Herbrecht R, Letscher-Bru V, Oprea C, Lioure B, Waller J, Campos F, Villard O (2002). Aspergillus galactomannan detection in the diagnosis of invasive aspergillosis in cancer patients. J Clin Oncol.

[33] Steinbach WJ, Addison RM, McLaughlin L, Gerrald Q, Martin PL, Driscoll T (2007). Prospective Aspergillus galactomannan antigen testing in pediatric hematopoietic stem cell transplant recipients. Pediatr Infect Dis.

[34] Smith PB, Benjamin DK, Alexander BD, Johnson MD, Finkelman MA, Steinbach WJ (2007). Quantification of 1,3-β-D-glucan levels in children: preliminary data for diagnostic use of the β-glucan assay in a pediatric setting. Clin Vaccine Immunol.

[35] Pfaller MA, Wolk DM, Lowery TJ (2015) T2MR and T2Candida: novel technology for the rapid diagnosis of candidemia and invasive candidiasis. Future Microbiol. [Epub ahead of print] 10.2217/fmb.15.111 26371384

[36] Pemán J, Zaragoza R (2010). Current diagnostic approaches to invasive candidiasis in critical care settings. Mycoses.

[37] Kim JH, Kang BC, Lee JH, Jang YJ, Lee BJ, Chung YS (2015). The prognostic value of gadolinium-enhanced magnetic resonance imaging in acute invasive fungal rhinosinusitis. J Infect.

[38] Yen TY, Huang LM, Lee PI, Lu CY, Shao PL, Chang LY (2011). Clinical characteristics of hepatosplenic fungal infection in pediatric patients. J Microbiol Immunol Infect.

[39] Fuster D, Tomás X, Granados U, Soriano A (2015). Prospective comparison of whole-body (18)F-FDG PET/CT and MRI of the spine in the diagnosis of haematogenous spondylodiscitis: response to comments by Soussan. Eur J Nucl Med Mol Imaging.

[40] Starkey J, Moritani T, Kirby P (2014). MRI of CNS fungal infections: review of aspergillosis to histoplasmosis and everything in between. Clin Neuroradiol.

[41] Caillot D, Couaillier JF, Bernard A, Casasnovas O, Denning DW, Mannone L (2001). Increasing volume and changing characteristics of invasive pulmonary aspergillosis on sequential thoracic computed tomography scans in patients with neutropenia. J Clin Oncol.

[42] Allan BT, Patton D, Ramsey NK, Day DL (1988). Pulmonary fungal infections after bone marrow transplantation. Pediatr Radiol.

[43] Taccone A, Occhi M, Garaventa A, Manfredini L, Viscoli C (1993). CT of invasive pulmonary aspergillosis in children with cancer. Pediatr Radiol.

[44] Thomas KE, Owens CM, Veys PA, Novelli V, Costoli V (2003). The radiological spectrum of invasive aspergillosis in children: a 10-year review. Pediatr Radiol.

[45] Thomas KE, Owens CM, Veys PA, Novelli V, Costoli V (2003). The radiological spectrum of invasive aspergillosis in children: a 10-year review. Pediatr Radiol.

[46] Demirkazik FB, Akin A, Uzun O, Akpinar MG, Ariyürek MO (2008). CT findings in immunocompromised patients with pulmonary infections. Diagn Interv Radiol.

[47] Signore A, Glaudemans AW (2011). The molecular imaging approach to image infections and inflammation by nuclear medicine techniques. Ann Nucl Med.

[48] Glaudemans AWJM. (2014) Nuclear medicine strategies to image infectious and inflammatory diseases. Dissertation, University of Groningen

[49] Sharma P, Mukherjee A, Karunanithi S, Bal C, Kumar R (2014). Potential role of 18F-FDG PET/CT in patients with fungal infections. AJR Am J Roentgenol.

[50] Hot A, Maunoury C, Poiree S, Lanternier F, Viard JP, Loulergue P (2011). Diagnostic contribution of positron emission tomography with [18F]fluorodeoxyglucose for invasive fungal infections. Clin Microbiol Infect.

[51] Bleeker-Rovers CP, Warris A, Drenth JP, Corstens FH, Oyen WJ, Kullberg BJ (2005). Diagnosis of Candida lung abscesses by 18F-fluorodeoxyglucose positron emission tomography. Clin Microbiol Infect.

[52] Avet J, Granjon D, Prevot-Bitot N, Isnardi V, Berger C, Stephan JL (2009). Monitoring of systemic candidiasis by 18F-FDG PET/CT. Eur J Nucl Med Mol Imaging.

[53] Kono M, Yamashita H, Kubota K, Kano T, Mimori A (2015). FDG PET imaging in Pneumocystis Pneumonia. Clin Nucl Med.

[54] Vahid B, Wildemore B, Nguyen C, Sistrun N, Marik PE (2006). Pulmonary blastomycosis masquerading as metastatic disease in the lung: a case report. Med Gen Med.

[55] Dang CJ, Li YJ, Zhan FH, Shang XM (2012). The appearance of pulmonary mucormycosis on FDG PET/CT. Clin Nucl Med.

[56] O’Doherty MJ, Barrington SF, Campbell M, Lowe J, Bradbeer CS (1997). PET scanning and the human immunodeficiency virus-positive patient. J Nucl Med.

[57] Liu Y, Wu H, Huang F, Fan Z, Xu B (2013). Utility of 18F-FDG PET/CT in diagnosis and management of mucormycosis. Clin Nucl Med.

[58] Reyes N, Onadeko OO, Luraschi-Monjagatta Mdel C, Knox KS, Rennels MA, Walsh TK, Ampel NM (2014). Positron emission tomography in the evaluation of pulmonary nodules among patients living in a coccidioidal endemic region. Lung.

[59] Wang J, Ju HZ, Yang MF (2014). Pulmonary cryptococcosis and cryptococcal osteomyelitis mimicking primary and metastatic lung cancer in (18)F-FDG PET/CT. Int J Infect Dis.

[60] Croft DR, Trapp J, Kernstine K, Kirchner P, Mullan B, Galvin J, Peterson MW (2002). FDG-PET imaging and the diagnosis of non-small cell lung cancer in a region of high histoplasmosis prevalence. Lung Cancer.

[61] Hamerschlak N, Pasternak J, Wagner J, Perini GF (2012). Not all that shines is cancer: pulmonary cryptococcosis mimicking lymphoma in [(18)] F fluoro-2-deoxy-d-glucose positron emission tomography. Einstein (Sao Paulo).

[62] Igai H, Gotoh M, Yokomise H (2006). Computed tomography (CT) and positron emission tomography with [18F]fluoro-2-deoxy-d-glucose (FDG-PET) images of pulmonary cryptococcosis mimicking lung cancer. Eur J Cardiothorac Surg.

[63] Salhab KF, Baram D, Bilfinger TV (2006). Growing PET positive nodule in a patient with histoplasmosis: case report. J Cardiothorac Surg.

[64] Ozsahin H, von Planta M, Müller I, Steinert HC, Nadal D, Lauener R (1998). Successful treatment of invasive aspergillosis in chronic granulomatous disease by bone marrow transplantation, granulocyte colony-stimulating factor-mobilized granulocytes, and liposomal amphotericin-B. Blood.

[65] Franzius C, Biermann M, Hülskamp G, Frosch M, Roth J, Sciuk J (2001). Therapy monitoring in aspergillosis using F-18 FDG positron emission tomography. Clin Nucl Med.

[66] Theobald I, Fischbach R, Hülskamp G, Franzius C, Frosch M, Roth J, Heindel W (2002). Pulmonary aspergillosis as initial manifestation of septic granulomatosis (chronic granulomatous disease, CGD) in a premature monozygotic female twin and FDG-PET diagnosis of spread of the disease. Radiologe.

[67] Camus V, Edet-Sanson A, Bubenheim M, Hitzel A, Becker S, David M (2015). ^18^F-FDG-PET/CT Imaging in patients with Febrile Neutropenia and Haematological Malignancies. Anticancer Resn.

[68] Wilkinson MD, Fulham MJ, McCaughan BC, Constable CJ (2003). Invasive aspergillosis mimicking stage IIIA non-small-cell lung cancer on FDG positron emission tomography. Clin Nucl Med.

[69] Sonet A, Graux C, Nollevaux MC, Krug B, Bosly A, Vander Borght T (2007). Unsuspected FDG-PET findings in the follow-up of patients with lymphoma. Ann Hematol.

[70] Nishikawa T, Kagawa T, Matsumi Y, Fujiwara T, Kataoka K, Matsuura M (2011). Lung cancer associated with pulmonary aspergillosis in the nodule of old mycobacterial infection. Kyobu Geka.

[71] Ahn BC, Lee SW, Lee J, Kim C (2011). Pulmonary aspergilloma mimicking metastasis from papillary thyroid cancer. Thyroid.

[72] Baxter CG, Bishop P, Low SE, Baiden-Amissah K, Denning DW (2011). Pulmonary aspergillosis: an alternative diagnosis to lung cancer after positive [18F]FDG positron emission tomography. Thorax.

[73] Xu B, Shi P, Wu H, Guo X, Wang Q, Zhou S (2010). Utility of FDG PET/CT in guiding antifungal therapy in acute leukemia patients with chronic disseminated candidiasis. Clin Nucl Med.

[74] Kim JY, Yoo JW, Oh M, Park SH, Shim TS, Choi YY, Ryu JS (2013). (18)F-fluoro-2-deoxy-d-glucose positron emission tomography/computed tomography findings are different between invasive and noninvasive pulmonary aspergillosis. J Comput Assist Tomogr.

[75] Bleeker-Rovers CP, Vos FJ, Wanten GJ, van der Meer JW, Corstens FH, Kullberg BJ, Oyen WJ (2005). 18F-FDG PET in detecting metastatic infectious disease. J Nucl Med.

[76] Tibúrcio FR, de Sá Rodrigues KE, Vasconcelos HM, Miranda DM, Simões e Silva AC (2015). Usefulness of positron emission tomography in the differentiation between tumor and infectious lesions in pediatric oncology: a case report. BMC Pediatr.

[77] Altinmakas E, Guo M, Kundu UR, Habra MA, Ng C (2015). Computed tomography and (18)F-fluorodeoxyglucose positron emission tomography/computed tomography findings in adrenal candidiasis and histoplasmosis: two cases. Clin Imaging.

[78] Karunanithi S, Kumar G, Sharma SK, Jain D, Gupta A, Kumar R (2015). Staging and response of sternal histoplasmosis by 18F-FDG PET/CT. Clin Nucl Med.

[79] Nakazato T, Mihara A, Sanada Y, Suzuki K, Aisa Y, Iwabuchi M, Kakimoto T (2010). Pneumocystis jiroveci pneumonia detected by FDG-PET. Ann Hematol.

[80] Miyazaki Y, Nawa Y, Nakase K, Kohashi S, Kadohisa S, Hiraoka A (2011). FDG-PET can evaluate the treatment for fungal liver abscess much earlier than other imagings. Ann Hematol.

[81] Roux S, Ferry T, Chidiac C, Bouaziz A, Ninet J, Pérard L (2014). Infectious thoracic aortic aneurysms: 7 cases and literature review. Rev Med Interne.

[82] Wallner M, Steyer G, Krause R, Gstettner C, von Lewinski D (2013). Fungal endocarditis of a bioprosthetic aortic valve. Pharmacological treatment of a Candida parapsilosis endocarditis. Herz.

[83] Morooka M, Ito K, Kubota K, Minamimoto R, Shida Y, Hasuo K, Ito T, Tasato D, Honda H, Teruya K, Kikuchi Y, Ohtomo K (2010). Whole-body 18F-fluorodeoxyglucose positron emission tomography/computed tomography images before and after chemotherapy for Kaposi sarcoma and highly active antiretrovirus therapy. Jpn J Radiol..

[84] Shrikanthan S, Aydin A, Dhurairaj T, Alavi A, Zhuang H (2005). Intense esophageal FDG activity caused by Candida infection obscured the concurrent primary esophageal cancer on PET imaging. Clin Nucl Med.

[85] Teyton P, Baillet G, Hindié E, Filmont JE, Sarandi F, Toubert ME (2009). Hepatosplenic candidiasis imaged with F-18 FDG PET/CT. Clin Nucl Med.

[86] Hanson MW, Glantz MJ, Hoffman JM, Friedman AH, Burger PC, Schold SC (1991). FDG-PET in the selection of brain lesions for biopsy. J Comput Assist Tomogr.

[87] Padma S, Sreehar S (2014). 18F FDG PET/CT identifies unsuspected bilateral adrenal histoplasmosis in an elderly immuno compromised patient. Indian J Med Res.

[88] Kasaliwal R, Malhotra G, Bukan A, Asopa RV, Wanjare S, Shah NS (2014). 18F-FDG PET as a monitoring tool to assess treatment response in bilateral adrenal histoplasmosis. Clin Nucl Med.

[89] Tsai YJ, Lin YH, Hsu CH, Yeh SD (2013). 18F-fluorodeoxyglucose positron emission tomography for the initial evaluation and monitoring of therapeutic response in bilateral adrenal histoplasmosis. Clin Imaging.

[90] Umeoka S, Koyama T, Saga T, Higashi T, Ito N, Kamoto T (2005). 18F-fluorodeoxyglocose uptake in adrenal histoplasmosis; a case report. Eur Radiol.

[91] Mackie GC, Pohlen JM (2005). Mediastinal histoplasmosis: F-18 FDG PET and CT findings simulating malignant disease. Clin Nucl Med.

[92] Dubbioso R, Pappatà S, Quarantelli M, D’Arco F, Manganelli F, Esposito M, Santoro L (2013). Atypical clinical and radiological presentation of cryptococcal choroid plexitis in an immunocompetent woman. J Neurol Sci.

[93] Vos FJ, Bleeker-Rovers CP, Oyen WJ (2013). The use of FDG-PET/CT in patients with febrile neutropenia. Semin Nucl Med.

[94] Chamilos G, Macapinlac HA, Kontoyiannis DP (2008). The use of 18F-fluorodeoxyglucose positron emission tomography for the diagnosis and management of invasive mould infections. Med Mycol.

[95] Ritz N, Ammann RA, Aebischer CC, Gugger M, Jaton K, Schmid RA, Aebi C (2005). Failure of voriconazole to cure disseminated zygomycosis in an immunocompromised child. Eur J Pediatr.

[96] Ho AY, Pagliuca A, Maisey MN, Mufti GJ (1998). Positron emission scanning with 18-FDG in the diagnosis of deep fungal infections. Br J Haematol.

[97] Go KG, Pruim TH, Que TH, Vaalburg W, Haaxma-Reiche H (2000). Evaluation of dissemination studies with FDG whole-body positron emission tomography in patients with suspected metastatic tumours of brain and spine. Acta Neurochir.

[98] Eubank WB, Mankoff DA, Takasugi J, Vesselle H, Eary JF, Shanley TJ (2001). 18fluorodeoxyglucose positron emission tomography to detect mediastinal or internal mammary metastases in breast cancer. J Clin Oncol.

[99] Kawabe J, Okamura T, Koyama K, Shakudo M, Sakamoto H, Kobashi T (1998). Relatively high F-18 fluorodeoxyglucose uptake in paranasal sinus aspergillosis: a PET study. Ann Nucl Med.

[100] Hanson MW, Glantz MJ, Hoffman JM, Friedman AH, Burger PC, Schold SC, Coleman RE (1991). FDG-PET in the selection of brain lesions for biopsy. J Comput Assist Tomogr.

[101] Glaudemans AW, Quintero AM, Signore A (2012). PET/MRI in infectious and inflammatory diseases: will it be a useful improvement?. Eur J Nucl Med Mol Imaging.

[102] de Jonge F, van Rheenen R (2015). Sunny and Tim: Wil jij fotodokter helpen?.

[103] Lupetti A, de Boer MG, Erba P, Campa M, Nibbering PH (2011). Radiotracers for fungal infection imaging. Med Mycol.

[104] Fischman AJ, Alpert NM, Livni E, Ray S, Sinclair I, Elmaleh DR, Weiss S, Correia JA, Webb D, Liss R (1991). Pharmacokinetics of 18F-labeled fluconazole in rabbits with candidal infections studied with positron emission tomography. J Pharmacol Exp Ther.

[105] Petrik M, Vlckova A, Novy Z, Urbanek L, Haas H, Decristoforo C (2015). Selected ^68^Ga-siderophores versus ^68^Ga-colloid and ^68^Ga-citrate: biodistribution and small animal imaging in mice. Biomed Pap Med Fac Univ Palacky Olomouc Czech Repub.

[106] Kumar V, Boddeti DK, Evans SG, Angelides S (2012). 68 Ga-Citrate-PET for diagnostic imaging of infection in rats and for intra-abdominal infection in a patient. Curr Radiopharm.

[107] Kumar V, Boddeti DK (2013). (68)Ga-radiopharmaceuticals for PET imaging of infection and inflammation. Recent Results Cancer Res.

[108] Siaens R, Eijsink VG, Vaaje-Kolstad G, Vandenbulcke K, Cornelissen B, Cuvelier C, Dierckx R, Slegers G (2006). Synthesis and evaluation of a 99mTechnetium labeled chitin-binding protein as potential specific radioligand for the detection of fungal infections in mice. Q J Nucl Med Mol Imaging.

[109] Siaens R, Eijsink VG, Dierckx R, Slegers G (2004). [123]I-Labeled chitinase as specific radioligand for in vivo detection of fungal infections in mice. J Nucl Med.

[110] Vos FJ, Donnelly JP, Oyen WJ, Kullberg BJ, Bleeker-Rovers CP (2012). 18F-FDG PET/CT for diagnosing infectious complications in patients with severe neutropenia after intensive chemotherapy for haematological malignancy or stem cell transplantation. Eur J Nucl Med Mol Imaging.

